# Parasitological and histopathological studies to the effect of aqueous extract of *Moringa oleifera* Lam. leaves combined with praziquantel therapy in modulating the liver and spleen damage induced by *Schistosoma mansoni* to male mice

**DOI:** 10.1007/s11356-022-23098-2

**Published:** 2022-09-28

**Authors:** Marwa I. Saad El-Din, Heba N. Gad EL-Hak, Mahi A. Ghobashy, Ranwa A. Elrayess

**Affiliations:** Zoology Department, Faculty of Science, Suez Canal University, Ismailia, Egypt

**Keywords:** *Schistosoma mansoni*, *Praziquantel*, *Moringa oleofera*, Liver, Spleen, Nuclear factor kappa β

## Abstract

This study assessed the effectiveness of an aqueous extract of *Moringa Oleifera* Lam. leaves (MOL) alone or in combination with praziquantel (PZQ) drug targeting–infected mice with *Schistosoma mansoni-*induced liver and spleen damage. Mice were divided into eight groups control mice group treated orally with saline. PZQ group: non-infected mice treated orally with 300 mg/kg bwt PZQ three consecutive days. MOL group: non-infected mice treated orally with 150 mg/kg bwt MOL extract for 15 days. PZQ/ MOL group: non-infected mice treated orally with 300 mg/kg bwt PZQ for three consecutive days and 150 mg/kg bwt *MOL* extract for 15 days. IF group: infected mice with 100 cercariae/mouse of the Egyptian strain of *S. mansoni*. IF/PZQ group infected mice with *S. mansoni* cercariae and treated orally with 300 mg/kg bwt PZQ for three consecutive days. IF/MOL group: infected mice with S. *mansoni* cercariae treated orally with 150 mg/kg bwt *MOL* extract for 15 days. IF/PZQ +MOL group: infected mice with S. *mansoni* cercariae treated orally with 300 mg/kg bwt PZQ for three consecutive days and 150 mg/kg bwt MOL extract for 15 days. Blood, liver, spleen, worm, and eggs were collected at the end of the experimental period. Treatment of infected mice with MOL and PZQ together significantly reduced the number of ova/g tissue and eliminated the parasites. In addition, the liver and spleen of infected mice showed less histopathological alteration and immunohistochemical expression of nuclear factor kappa β (NF-Kβ). We can conclude that MOL extract combined with PZ has a curative effect on *S. mansoni* infection and helped to lessen its pathological effects.

## Introduction

The parasitic trematode flatworm Schistosoma causes schistosomiasis (Chuah et al. 2019). Freshwater snails serve as intermediate hosts, releasing parasitic worm larvae that enter the skin and contaminate the water. The intestines, liver, kidneys, and blood vessels all contain mature infectious larvae that can reproduce (Verjee 2019). Released egg antigens that are entrapped in organ tissues cause clinical disease by inducing granulomatous reactions involving T cells, macrophages, and eosinophils. The number and location of trapped eggs affect symptoms and signs (McManus et al. 2020). Initial inflammatory responses to schistosoma are reversible, and the disease’s later stages are more incapacitating (Stadecker et al. 2004). Organ fibrosis is increased by pathological collagen deposition and may not fully reverse (Wynn 2008). Up to 25% more people can die as a result of acute schistosomiasis (Verjee 2019). The main health problems induced with schistosomiasis are malignancy, cardiovascular, hepatic, pulmonary, renal, and neurological diseases (Chen 2014). Schistosomiasis results in histological changes in various organs, including the lungs, liver, kidney, spleen, and intestine. In schistosomiasis, the host immune system responded to some eggs that were trapped in tissues by inflaming, granulomatous, and fibrotic reactions (Wilson et al. 2007). Schistosomiasis frequently takes time to manifest, delaying diagnosis (Shebel et al. 2012). Acute schistosomiasis patients typically show symptoms four to 8 weeks after exposure to infected water (Visser et al. 1995).

Schistosomiasis can be treated with the two anthelmintic drugs praziquantel (PZQ) and oxamniquine (Cioli et al. 2014). Two anthelmintic drugs work to treat the illness, reduce morbidity, and then stop the spread of the parasite in endemic areas (Sturrock 2001). PZQ is the preferred medication for schistosomiasis mass community treatment, but it is less effective against juvenile cases (Vale et al. 2017). PZQ has a cure rate of 65–90% after a single oral dose, it is the least expensive medication that is also the most effective (Verjee 2019). Through tegument vacuolation, its pharmacological action alters the parasite’s membrane permeability. Developing schistosomula are more resistant to the host immune system’s attacks than mature worms, which allows the infection to persist (Xiao et al. 2018). Resistance to PZQ is also clearly defined. Following the start of treatment, mild side effects from the reactions of dying worms include dizziness, headache, nausea, vomiting, diarrhea, abdominal pain, bloody stool, urticaria, and fever (Sousa-Figueiredo et al. 2012; Mengarda et al. 2022). However, PZQ efficacy decreased as drug-resistant strains emerged as a result of widespread drug administration directed at specific populations, which predicted the selection of drug-resistant strains (Vale et al. 2017; Amara and Saadawi 2022). There are many factors besides drug resistance that can contribute to PZQ therapy failure, such as high rates of PZQ-resistance transmission and development (Geerts and Gryseels 2000). PZQ was regarded as a genotoxic, carcinogenic, and hepatotoxic drug (Omar et al. 2005), and the increasing dose was inappropriate because it had slight effect on fibrosis (Ditteová et al. 2003). Thus, PZQ combination even with the natural plant could be helpful to overcome the PZQ major weakness and side effects (Gouveia et al. 2018).

*Moringa oleifera* showed promise in controlling schistosomiasis due to its bioactive compounds that are toxic to some species of animals (Almanzor et al. 2014). *M. oleifera* had been found to have anthelmintic properties (Cabardo Jr and Portugaliza 2017). Paikra (2017) revealed that anthelmintic properties have been noted in the leaves, flowers, and pods. Along with other plants, *M. oleifera* was used to treat helminths in greater effectiveness than other plants (Cabardo Jr and Portugaliza 2017). Phytochemical analysis revealed that aqueous extracts of *M. oleifera* were found to contain saponin, steroids, carbohydrates, alkaloids, tannins, proteins, flavonoids antioxidants, antimicrobial alkaloids, lectin, and trypsin inhibitors (Patel et al. 2014). These compounds may paralyze and kill helminths worm (Mukadam 2016). A little exploration of the aqueous extract of *M. oleifera* leaves potential effects for schistosomiasis found. Due to its record of anthelminthic, antioxidant, and anti-inflammatory properties, it becomes a challenge to evaluate the potential efficacy of the aqueous extract of *M. oleifera* leaves combined with PZQ therapy in modulating the liver and spleen damage induced by *Schistosoma mansoni* to male mice.

## Materials and methods

### Experimental materials

#### *Moringa oleifera* (MOL)

##### Preparation of MOL aqueous extract

MOL was obtained from a local market in Egypt. Aqueous extracts were used freshly prepared. One hundred grams of powdered *M. oleifera* leaves (1:10 w/v) were extracted by maceration (24 h) on tap water at room temperature (25–30 °C) according to Bozinou et al. (2019).

#### Qualitative analysis of MOL with HPLC instrument

HPLC analysis was performed on Waters 2690 Alliance HPLC system equipped with a Waters 996 photodiode array detector. A 200 mg/ml extract of was dissolved and sonicated for 30 min filtered using a 0.22-μm nylon syringe filter, then 10 μl was injected. HPLC analysis conditions with Column C18 Inertsil ODS 4.6×250mm, 5μm, mobile phase: 0.1% phosphoric acid in water: acetonitrile, mode of elution: gradient, flow rate: 1ml/min, temperature: ambient and wavelength: 280 nm. The stock solution of 10 different standards (gallic acid; catechin; chlorogenic acid; rutin; ellagic acid; caffeic acid; hesperidin; quercetin; kampeferol and apigenin) in methanol was prepared. Each of the standards was filtered using a 0.22-μm syringe filter, then 10 μl were injected. The peaks were identified by comparing the retention time.

#### Praziquantel (PZQ)

PZQ was produced by SEDCO pharmaceutical Co. on 6 October City—Egypt. Each 600 mg tablet was broken down into a fine white powder and put in 5 mL of distilled water for suspension.

### Experimental animals and their mode of infection

Fifty-six male albino CD-1 mice weighing between (18–20 g) were procured from the Schistosome Biological Supply Program, Theodor Bilharz Research Institute (SBSP, and TBRI) in Giza, Egypt. The animals were kept in standard caging circumstances, which included a temperature of 21 ± 1°C and free access to water and pellet meal and cages cleaned every 3 days daily. All studies were followed the Egyptian guidelines for the care and use of laboratory animals. Mice were infected by injecting with Schistosoma *mansoni* cercariae subcutaneously. Cercariae were obtained from the infected *Biomphalaria Alexandrina* snails, from SBSP, and TBRI. The number of cercariae was determined by using a dissecting microscope. Generally, three counts were made, and the average was used to calculate the number of cercariae per 0.1 ml of the cercarial suspension. Infection was done subcutaneously with 100 cercariae/mouse of the Egyptian strain of *S. mansoni*. The infected and uninfected mice were left 40 days. Mice left from 35 to 40 days for schistosoma maturation and mating (Kadji Fassi et al. 2022). The mice were weighed weekly. The experiment design was approved by the Ethical Committee of the Faculty of Science Suez Canal University Egypt and followed ARRIVE guidelines.

### Experimental design

Mice were divided into eight groups (7 mice each) as following:

Control mice group treated orally with saline. PZQ group: non-infected mice treated orally with 300 mg/kg bwt PZQ three consecutive days. MOL group: non-infected mice treated orally with 150 mg/kg bwt *MOL* extract for 15 days. PZQ/MOL group: non-infected mice treated orally with 300 mg/kg bwt PZQ for three consecutive days and 150 mg/kg bwt *MOL* extract for 15 days. IF group: infected mice with S. *mansoni* cercariae. IF/PZQ group infected mice with *S. mansoni* cercariae and treated orally with 300 mg/kg bwt PZQ for three consecutive days. IF/MOL group: infected mice with S. *mansoni* cercariae treated orally with 150 mg/kg bwt MOL extract for 15 days. IF/PZQ +MOL group: infected mice with S. *mansoni* cercariae treated orally with 300 mg/kg bwt PZQ for three consecutive days and 150 mg/kg bwt *MOL* extract for 15 days.

#### Blood sampling

Blood samples were collected at the end of the experimental period on day 55 from heart puncture in a test tube without anticoagulant. The samples were put in an inclined position for 20 min at room temperature, and then put in refrigerator, then centrifuged at 3000 rpm for 10 min and the clear serum was collected carefully and stored at −20°C until estimating the liver function.

#### Worm count and percentage of reduction

Isoflurane anesthesia was used to produce rapid loss of consciousness without pain (Miller et al. 2015). Mice were decapitated and their bodies were washed with tap water to remove any adherent hairs. The abdominal muscles and peritoneum were dissected to expose the internal organ. The liver and spleen were weighed. The liver was soaked in a 10% natural saline solution where flukes were removed and counted from the hepato-portal veins using dissecting needles under the stereomicroscope. Adult worms were counted and separated according to sex.

#### The number of Schistosoma mansonai eggs and the percentage of eggs at different developmental stages

The number of ova per gram of hepatic and small intestine tissue were count from infected mice (Domingo and Warren 1969). The percentage of eggs at different developmental stages in the small intestine and the liver was examined in three samples per animal and the mean of each stage per animal was obtained. Eggs were count and classified into their stages of development according to Cheever (1968) into viable immature eggs, viable mature and dead eggs.

#### Histopathological investigation

Liver and spleen samples from each animal were preserved in 10% buffered formalin solution, until dehydrated, sectioned and stained with hematoxylin and eosin (Bancroft and Stevens 1975) and Masson Trichrome (O'connor and Valle 1982). The number of granulomas in 5 successive fields (40×10) was counted and recorded.

#### Hepatic granuloma count and diameter measurement

Measurement of granuloma diameter was done only for non-contiguous granulomas, each containing a single egg in the center using a calibrated ocular micrometer. The mean granuloma diameter (M.G.D) was calculated by measuring two diameters of the lesion at right angles to each other (Mahmoud and Warren 1974).

#### Immunohistochemical staining of liver and spleen using Anti-nuclear factor kappa β (NF-Kβ)

Liver and spleen sections were placed over positively charged slides, then placed in 65°C oven for an hour. The sections were deparaffinized and rehydrated, washed, and submerged in Tris-buffered solution for pH adjustment. Sections were then incubated in 0.3% hydrogen peroxide (H_2_O_2_) at room temperature for 30 min to inhibit the activity of endogenous peroxidase. Then, slides were submerged in blocking solution (normal goat serum) at room temperature for 30 min to help antigen recovery and eliminate non-specific background staining, and then incubated with primary anti-body NFK-B (Santa Cruz Biotechnology Inc., Santa Cruz, CA, USA) at 1:300 concentrations room temperature for 60 min. The sections were washed with 0.1 M phosphate, 0.15 M NaCl; pH 7.5, and incubated with the secondary biotinylated antibody at room temperature in a humidity chamber for an hour, then incubated with avidin-biotin horseradish peroxidase complex for 30 min. The color reaction was developed when adding DAB solution (0.5 mg/ml DAB and 0.1% H_2_O_2_) to the sections for 10 min, after that, rinsed with distilled water. The sections were counterstained by hematoxylin for 2 min, dehydrated in graded alcohol and cleared by xylene. Eventually, coverslips were placed, and slides were examined under the light microscope. Fields were captured by a digital camera (Canon DSLR EOS 1200D, Japan) which was mounted on a light microscope (BX60, Olympus, Japan). The obtained images were transferred to the computer system for analysis. ImageJ software (Version 1.41a, NIH, USA) was used for the calculation of the percentage area analysis in the images of anti-NF-Kβ immune–stained sections. The areas of IHC positivity in the liver and spleen for eight random images of each slide were assessed in ImageJ software according to (Gad El-Hak et al. 2022).

#### Liver function investigation

Measurement of serum alanine aminotransferase (ALT) and aspartate aminotransferase (AST) were determined spectrophotometrically according to the method described by Reitman and Frankel (1957) using commercial kits ( Cayman company, USA catalog number 700260 and 701640) respectively. Total protein was estimated according to Okutucu et al. (2007) and globulin was estimated according to Goldenberg and Drewes (1971) using commercial kits (Cayman company, USA catalog number 701780 and MyBiosource USA catalog number MBS8309619).

### Statistical analysis

Statistics were calculated with SPSS for windows version 17.0, and the means value obtained in the different groups were compared by one-way ANOVA followed by Duncan’s. All results were expressed as mean values ± SE and significance was defined as *P*< 0.05.

## Results

### The qualitative analysis of MOL extract

HPLC of an aqueous MOL extract indicated the standard presence of caffeic acid Table 1.

**Table 1:** Qualitative analysis of *Moringa oleifera* leaves extract

Reference compounds	Detected/not detected
Gallic acid	Not detected
Catechin	Not detected
Chlorogenic acid	Not detected
Rutin	Not detected
Ellagic acid	Not detected
Caffeic acid	Detected
Hesperidin	Not detected
Quercetin	Not detected
Kampeferol	Not detected
Apigenin	Not detected

### The final weight and absolute body weight of the liver and spleen

The infected mice showed a significant decrease in the final body weight and increase in the liver and spleen absolute weight compared to the control group as shown in Table 2. Treatment with Moringa leaves extract and or praziquantel induced a significant increase in the final body weight and decrease in the liver and spleen weight of the infected treated animals when compared with that of infected control mice.

**Table 2:** Final body weight and absolute liver and spleen weight of *Schistosoma mansonai*-infected mice treated with praziquantel and/or aqueous extract of *Moringa oleifera* leaves

Treatment	Body weight (g)	Absolute organ weight (g)	
		Liver (g)	Spleen (g)
Control	24.285±1.01^a^	0.595±0.06^c^	0.059±0.02^b^
PZQ	15.142±0.70^cd^	0.778±0.10^abc^	0.21±0.14^ab^
MOL	25.71±0.68^a^	0.985±0.05^a^	0.268±0.12^ab^
PZQ/MOL	20.71±0.80^b^	0.66±0.08^b^	0.103±0.04^ab^
IF	13.57±0.29^d^	0.9152±0.08^a^	0.42±0.2^a^
IF/PZQ	15.28±0.60^cd^	0.632±0.05^bc^	0.137±0.04^ab^
IF/MOL	16.14±0.50^c^	0.838±0.09^abc^	0.143±0.05^ab^
IF/PZQ+MOL	20.017±1.09^b^	0.8560±0.07^ab^	0.158±0.05^ab^

Values are expressed as means ± SE (*n* = 7). Mean values with different superscript letters within the same row are significantly different at *P* ≤ 0.05 using ANOVA followed by Duncan’s multiple comparison test. *PZQ*, praziquantel; *MOL*, *Moringa oleifera* leave extract; *PZQ/MOL*, praziquantel and *Moringa oleifera* leave extract; *IF*, infected mice with *Schistosoma mansonai*; *IF/PZQ*, infected mice with *Schistosoma mansonai* treated with Praziquantel; *IF/MOL*, infected mice with *Schistosoma mansonai* treated with *Moringa oleifera* leave extract; *IF/PZQ+MOL*, infected mice with *Schistosoma mansonai* treated with praziquantel and *Moringa oleifera* leave extract

### The mean worm count and percentage of their reduction

The perfusion of the hepatic portal and mesenteric veins of infected mice treated with MOL, PZQ, or both revealed a significant reduction in the mean worm count compared to the infected group Table 3.

**Table 3:** The mean worm and egg count and the percentage of eggs at different developmental stages of *Schistosoma mansonai*-infected mice treated with praziquantel and/or aqueous extract of *Moringa oleifera* leaves

Treatment	Parameters					
	The mean worm count	The mean egg count		The percentage of eggs at different developmental stages		
		In liver	In intestine	% Immature ova	% Mature ova	% Dead ova
IF	10.71±1.4^a^	15872.57±914.9^a^	14129.5±496.5^a^	48.86±0.6^a^	46.14±0.7^a^	5.10±0.4^d^
IF/PZQ	1.66±0.5^b^	1842.86±181.3^b^	2020.57±129.1^b^	45.57±2.0^b^	42.43±1.9^b^	12.0±0.7^c^
IF/MOL	0.00±0.0^b^	2316.14±486.3^b^	2319.43±278.3^b^	0.00±0.00^c^	16.57±1.4^c^	83.4±1.4^b^
IF/PZQ+MOL	1.46±0.9^b^	1427.42±152.2^b^	2408.57±240.3^b^	0.00±0.00^c^	4.10±0.9^d^	96.0±0.9^a^

Values are expressed as means ± SE (*n* = 7). Mean values with different superscript letters within the same row are significantly different at *P* ≤ 0.05 using ANOVA followed by Duncan’s multiple comparison test. PZQ, praziquantel; MOL, *Moringa oleifera* leave extract; PZQ/MOL, praziquantel and *Moringa oleifera* leave extract; *IF*, infected mice with *Schistosoma mansonai*; *IF/PZQ*, infected mice with *Schistosoma mansonai* treated with praziquantel; *IF/MOL*, infected mice with *Schistosoma mansonai* treated with *Moringa oleifera* leave extract; *IF/PZQ+MOL*, infected mice with *Schistosoma mansonai* treated with praziquantel and *Moringa oleifera* leave extract

### The eggs

The mean egg load in hepatic and intestinal tissues of the infected untreated group showed a significant increase at *P*≤0.05 in the number of eggs when compared to the corresponding all the treated infected groups Table 3.

As presented in Table 3, the mean number of *S. mansoni* mature and immature eggs of infected mice significantly increased compared to the corresponding all the treated infected groups. The reduction rate of mature and immature eggs obtained from the infected groups of mice treated with PZQ or MOL or both was significant compared to the infected group. Dead eggs were detected increased in infected mice treated with PZQ or MOL Table 3.

### Liver function investigation

The effect of treatment on infected mice with PZQ and/or MOL on liver function was evaluated by measuring the serum levels of ALT, AST, total protein, and globulins shown in Table 4. *S. mansoni* infection exhibited elevation of serum ALT and AST and reduction of the total protein and globulin levels compared with the control group. Contrarily, the concomitant administration of PZQ and/or MOL significantly modulate the disturbance of the liver function induced in the infected group. Treatment with the combination of PZQ and MOL significantly restored serum ALT, AST, total protein, and globulin to concentrations nearest to those of the uninfected controls.

**Table 4:** Liver function in blood serum of *Schistosoma mansonai*-infected mice treated with praziquantel and/or aqueous extract of *Moringa oleifera* leaves

Treatment	Liver function parameter			
	Alanine aminotransferase (ALT) (U/l)	Aspartate aminotransferase (AST) (U/l)	Total protein (mg/dL)	Globulin (mg/dL)
Control	47.00±2.2^f^	84.28±4.8^b^	9.38±0.1^a^	2.89±0.2^a^
PZQ	86.2±1.3^b^	78.85±4.4^b^	5.24±0.5^c^	1.66±0.3^b^
MOL	64.00±1.4^d^	85.42±4.0^b^	8.46±0.1^ab^	2.52±0.2^a^
PZQ/MOL	72.28±1.8^c^	98.57±13.5^b^	5.24±0.4^c^	1.55±0.2^b^
IF	93.71±0.8^a^	172.28±13.1^a^	3.62±0.1^d^	1.31±0.2^b^
IF/PZQ	75.42±0.8^c^	171.28±12.4^a^	7.80±0.6^b^	1.70±0.3^b^
IF/MOL	74.85±2.7^c^	181.28±3.8^a^	5.34±0.3^c^	1.45±0.2^b^
IF/PZQ+MOL	55.28±0.7^e^	103.00±1.0^b^	7.34±0.2^b^	1.39±0.2^b^

Values are expressed as means ± SE (*n* = 5). Mean values with different superscript letters within the same row are significantly different at *P* ≤ 0.05 using ANOVA followed by Duncan’s multiple comparison test. *PZQ*, praziquantel; *MOL*, *Moringa oleifera* leave extract; *PZQ/MOL*, praziquantel and *Moringa oleifera* leave extract; *IF*, infected mice with *Schistosoma mansonai*; *IF/PZQ*, infected mice with *S. mansonai* treated with praziquantel; IF/MOL, infected mice with *Schistosoma mansonai* treated with *Moringa oleifera* leave extract; *IF/PZQ+MOL*, infected mice with *Schistosoma mansonai* treated with praziquantel and *Moringa oleifera* leave extract

### The histological examination of the liver and spleen

**Fig. 1:**
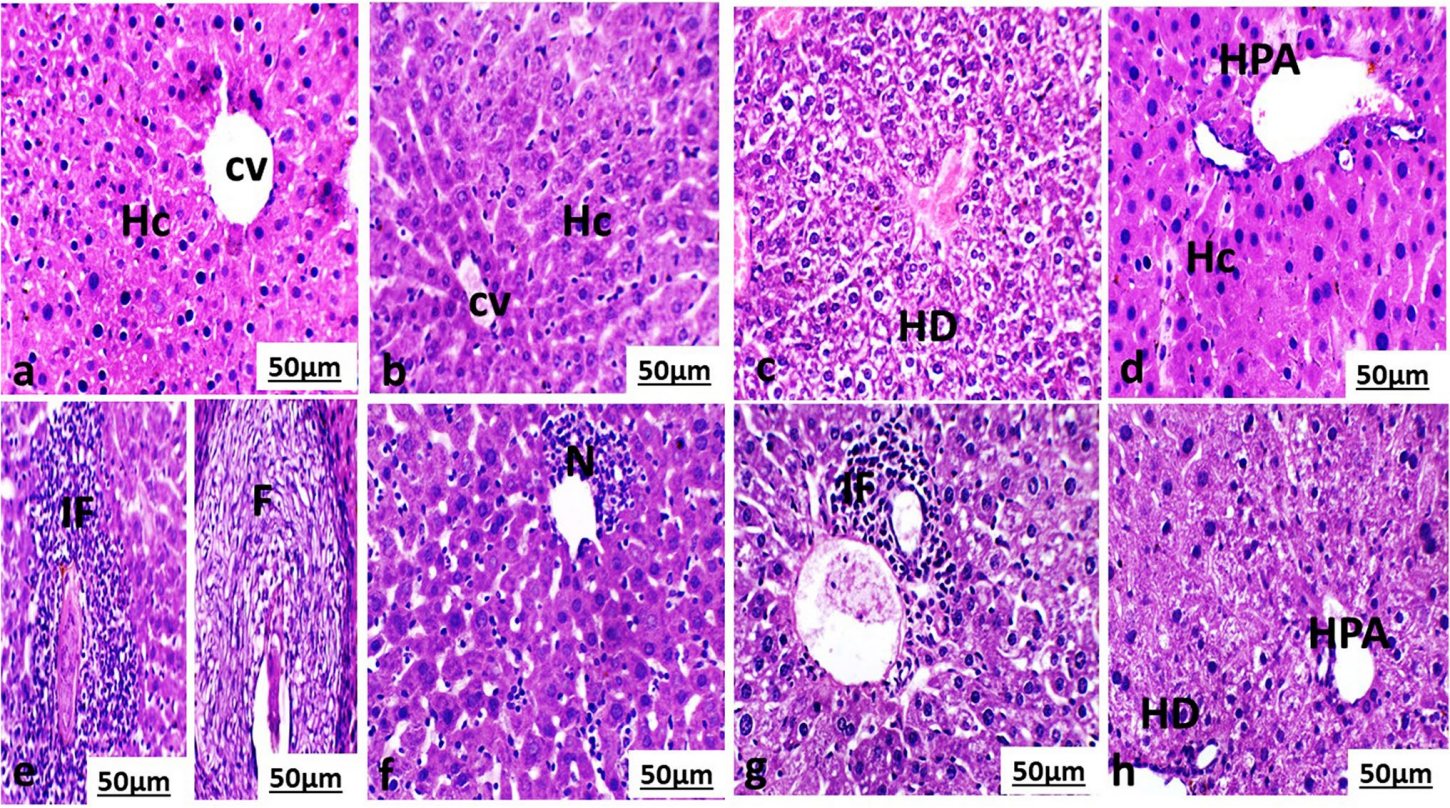
Photomicrograph of **a** liver section of a normal mouse and **b** liver section of the treated mouse with *Moringa oleifera* aqueous leaves extract (MOL) showed polyhedral hepatocytes (Hc) forming a network of hepatic strands around the central vein (cv). **c** Liver section of the treated mouse with praziquantel (PZQ) showed vacuolated hepatocytes (Hc) forming a network of hepatic strands surrounded congestion central vein (cv). **d** Liver section of the treated mouse with PZQ and MOL showed polyhedral hepatocytes (Hc) forming a network of hepatic strands surrounding the hepatic portal area (HPA). **e** Liver section of *S. mansoni*-infected, non-treated mouse showed hepatic lobular distortion and formation of granulomas with egg and worms surrounded by fibrotic cell (F) and inflammatory cell (F). **f** Liver section of *S. mansoni*-infected, treated mouse with MOL showed many necrotic foci with pyknotic nucleus (N) are observed among hepatocytes near the central vein. **g** Liver section of *S. mansoni*-infected, treated mouse with PZQ showed infiltration of inflammatory cells near the portal area. **h** Liver section of *S. mansoni*-infected, treated mouse with PZQ and MOL showed vacuolated hepatocytes (HD) near the portal area. (HE, 400×)

The histological changes of the liver of control, non-infected mice treated with MOL or PZQ or both were normal with mild histological alteration Fig. 1a, b, c, and d. The liver section of the control, non-infected mice treated with control, non-infected mice treated with MOL or both MOL and PZQ mice showed polyhedral hepatocytes forming a network of hepatic strands around the central vein. Each hepatocyte encloses a finely granulated cytoplasm with a round and centrally located nucleus. The liver section of treated mice with PZQ showed hydrobic degeneration

The liver of infected non-treated mice Fig. 1e showed granulomatous reaction surrounded by inflammatory cell or fibrous connective tissue.

The liver of infected mice treated with MOL or PZQ or both showed degenerative alterations to the hepatic structure Fig. 1f, g, and h. It was observed that liver cells were vacuolated and fewer necrotic foci were seen especially near the central vein. Some of these cells possessed a pyknotic nucleus. In addition to bile duct proliferation and periportal inflammation.

### Hepatic granuloma count and diameter measurement

**Fig. 2:**
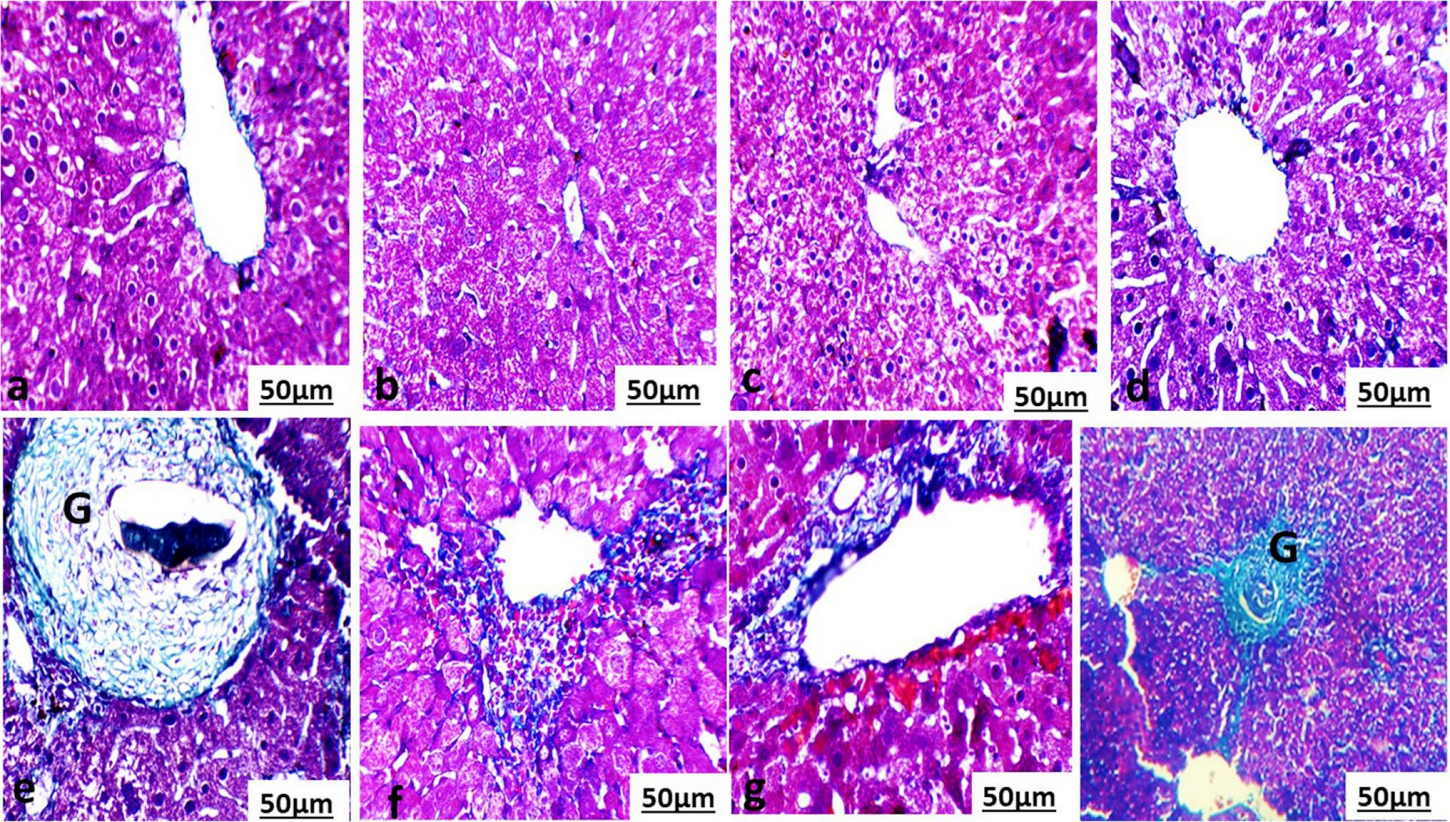
Photomicrograph of Liver section stained with Masson trichrome of **a** a normal mouse and **b** treated mouse with *Moringa oleifera* aqueous leaves extract (MOL) showed polyhedral hepatocytes forming a network of hepatic strands around the central vein. **c** Treated mouse with praziquantel (PZQ) showed vacuolated hepatocytes (Hc) forming a network of hepatic strands surrounding the central vein. **d** Treated mouse with PZQ and MOL showed polyhedral hepatocytes forming a network of hepatic strands around surrounding the central vein. **e** *S. mansoni*-infected, non-treated mouse showed hepatic lobular distortion and formation of granulomas (G) with egg surrounded by fibrotic cell. **f** *S. mansoni*-infected, treated mouse with MOL showed many necrotic foci with pyknotic nucleus are observed among hepatocytes near the central vein. **g** *S. mansoni*-infected, treated mouse with PZQ showed infiltration of inflammatory cells near the portal area. **h** Liver section of *S. mansoni*-infected, treated mouse with PZQ and MOL showed decreased granulomas (g) and fibrotic cells surrounding it relative to the infected untreated group. (400×)

The areas of liver section suffering from granuloma in the infected and treated groups are shown in Fig. 2. We noticed a central Schistosoma egg surrounded by infiltrated immunological cells and fibers. There was a significant reduction in granuloma number in the infected groups treated with PZQ or MOL or both as compared to the infected untreated animals. All the infected treated groups showed a significant decrease in the granuloma number and diameter as compared to the infected untreated group Table 5. The highest reduction rate in granuloma number and diameter was recorded in IF/PZQ+MOL group in comparison with the infected untreated group.

**Table 5:** Hepatic granuloma count and diameter measurement of *Schistosoma mansonai*-infected mice treated with praziquantel and/or aqueous extract of *Moringa oleifera* leaves

Treatment	No of granuloma in successive power fields (10×10)	Mean granuloma diameter in um	Types of granuloma cellular %	Types of granuloma fibro cellular%	State of eggs intact%	State of eggs degenerated %
Control	0±0.0	0±0.0	0±0.0	0±0.0	0±0.0	0±0.0
PZQ	0±0.0	0±0.0	0±0.0	0±0.0	0±0.0	0±0.0
MOL	0±0.0	0±0.0	0±0.0	0±0.0	0±0.0	0±0.0
PZQ/MOL	0±0.0	0±0.0	0±0.0	0±0.0	0±0.0	0±0.0
IF	15.43±4.9^a^	361±18.2^a^	85±2.6^a^	60±3.5^a^	97±3.9^a^	80±2.4^a^
IF/PZQ	6.2±2.64^c^	178±22.7^c^	57±2.7^c^	43±5.9^b^	60±3.2^c^	40±2.8^b^
IF/MOL	6.51±4.47^b^	221±17.2^b^	70±4.7^b^	30±2.6^c^	95±2.8^b^	5±1.7^c^
IF/PZQ+MOL	0.8±0.03^d^	154±12.2^d^	40±2.4^d^	15±1.5^d^	20±2.3^d^	3±0.9^d^

Values are expressed as means ± SE (*n* = 5). Mean values with different superscript letters within the same row are significantly different at *P* ≤ 0.05 using ANOVA followed by Duncan’s multiple comparison test. *PZQ*, praziquantel; *MOL*, *Moringa oleifera* leave extract; *PZQ/MOL*, praziquantel and *Moringa oleifera* leave extract; *IF*, infected mice with *Schistosoma mansonai*; *IF/PZQ*, infected mice with *Schistosoma mansonai* treated with praziquantel; *IF/MOL*, infected mice with *Schistosoma mansonai* treated with *Moringa oleifera* leave extract; *IF/PZQ+MOL*, infected mice with *Schistosoma mansonai* treated with praziquantel and *Moringa oleifera* leave extract

### The histological examination of the spleen

**Fig. 3:**
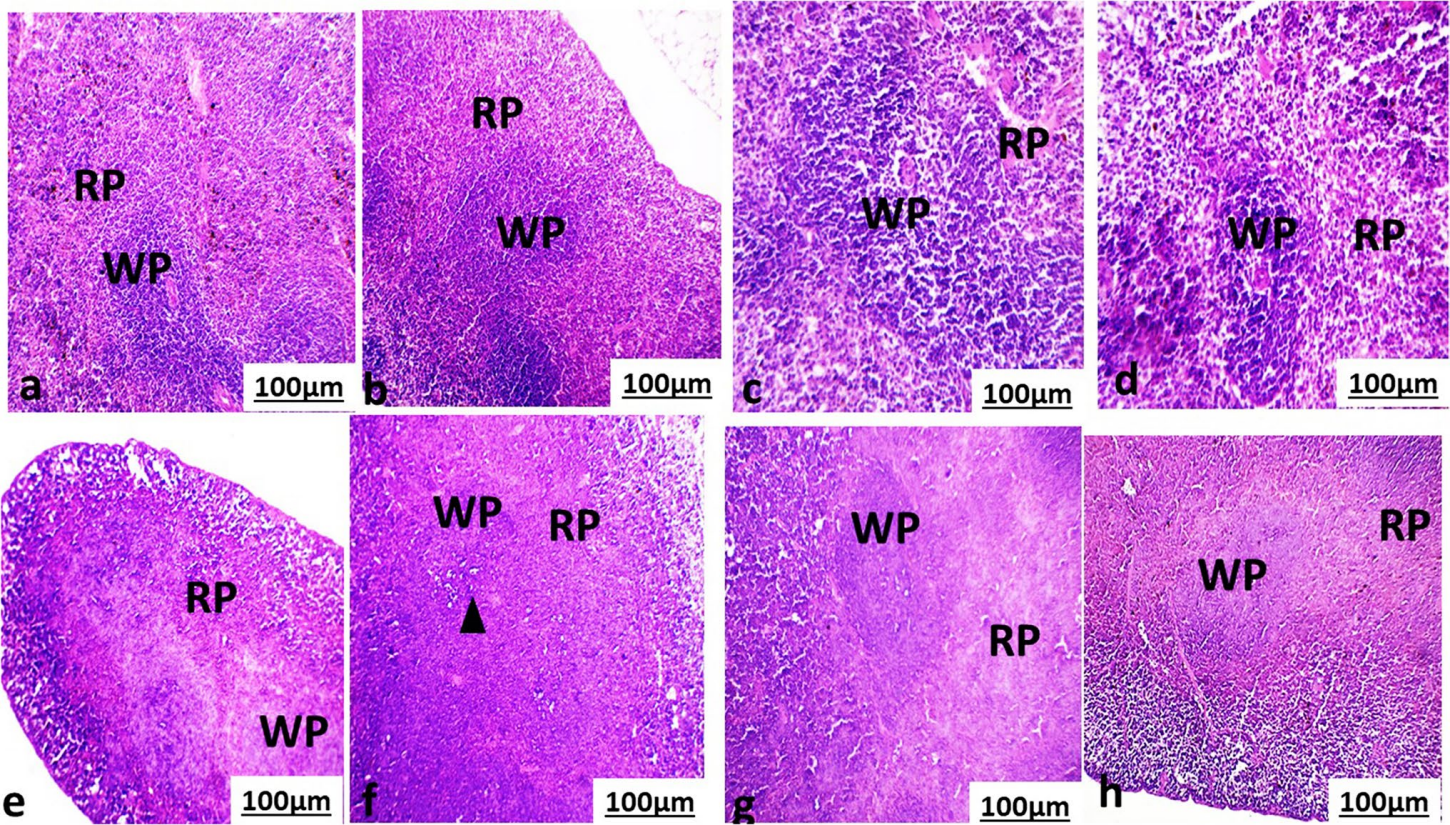
Photomicrograph of spleen section of **a** normal mouse and **b** not infected treated mouse with *Moringa oleifera* aqueous leaves extract (MOL). **c** Non-infected treated mouse with Praziquantel (PZQ). **d** Treated mouse with PZQ and MOL showed normal red (RP) and white pulps (WP). **e** *S. mansoni*-infected, non-treated mouse showed necrotic white (WP) and disorganized red pulp (RP). **f** *S. mansoni*-infected, treated mouse with *Moleifera* leave extract (MOL) showed decreased histological damage of Δwhite (WP) and red pulp (RP). **g** *S. mansoni*-infected, treated mouse with praziquantel (PZQ). **h** *S. mansoni*-infected, treated mouse with PZQ and MOL showed organized white pulp and red pulp with mild persist of necrotic white pulp. (HE, 100×)

The histological changes of the spleen of control, non-infected mice treated with MOL or PZQ or both showed normal histological structure Fig. 3a, b, c, and d. The spleen is composed mainly of red and white pulps. The white pulp is composed of a reactive germinal center with mantle zone and marginal zone with adjacent splenic arteriole. The red pulp with some sinus and cord appears in it.

The spleen of infected non-treated mice with Fig. 3e showed necrotic pale and disorganization and distorted of the white pulp with depletion of the lymphocyte.

The spleen of infected mice treated with MOL or PZQ or both Fig. 3f, g, and h showed a mild improvement in the histological structure of the spleen with the decrease of necrotic white pulp and recovery of the red pulp.

### Immunohistochemical staining of liver and spleen using Anti-nuclear factor kappa B (NFK-B)

**Fig. 4:**
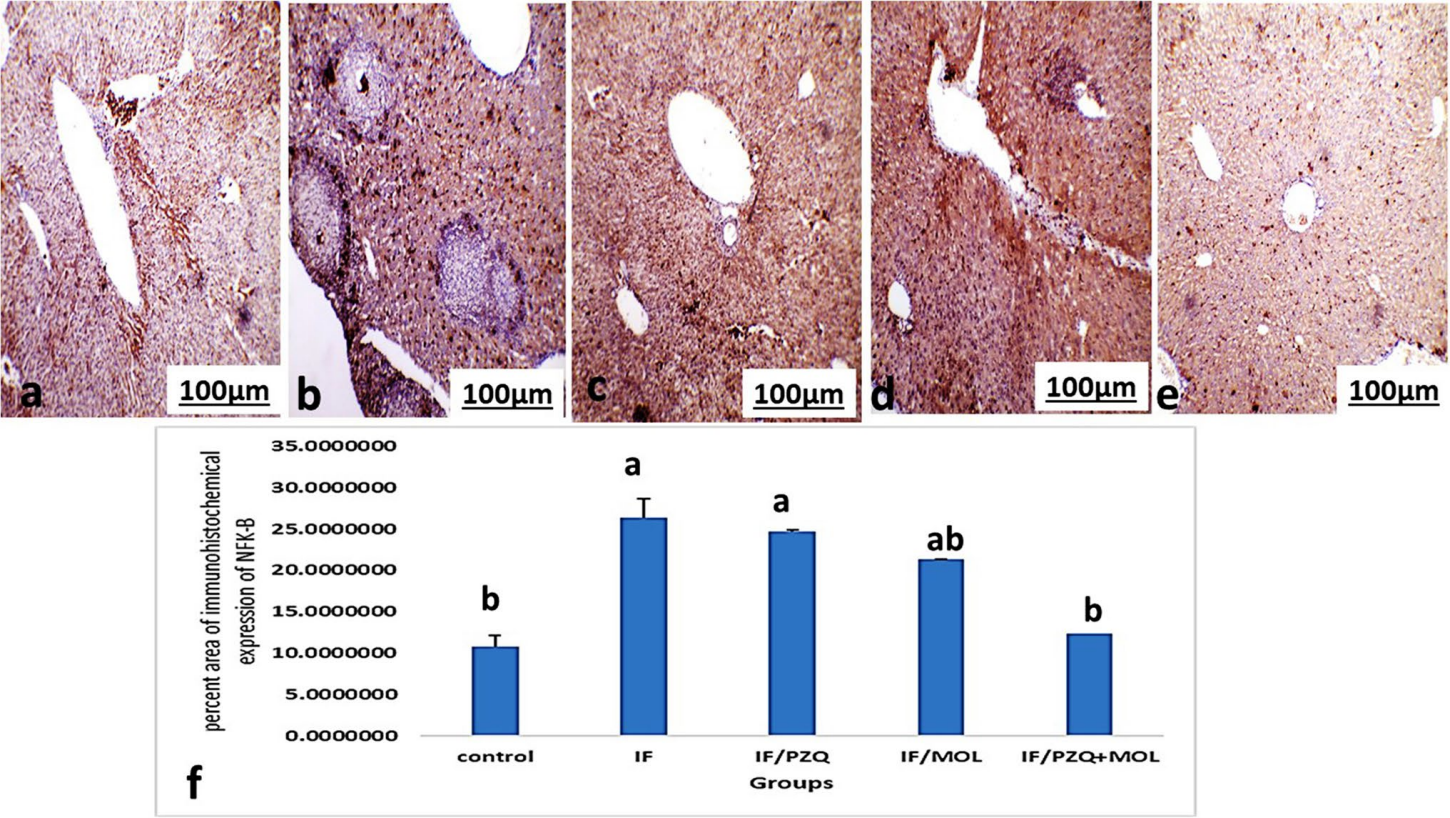
Representative immunohistochemical staining for nuclear factor kappa β (NF-κβ) in the liver of **a** normal mouse and **b** *S. mansoni*-infected, non-treated mouse. **c** *S. mansoni*-infected, treated mouse with *Moringa oleifera* leave extract (MOL). **d** *S. mansoni*-infected, treated mouse with praziquantel (PZQ). **e** *S. mansoni*-infected, treated mouse with PZQ and MOL (100×). **f** Histogram of the mean percentage areas of NF-κβ, in the hepatocytes of different groups (*n* = 5). Different superscript letters denote significant differences at *P* ≤ 0.05. IF, infected mice with *Schistosoma mansonai*; IF/PZQ, infected mice with *Schistosoma mansonai* treated with praziquantel; IF/MOL-infected mice with *S. mansonai* treated with *Moringa oleifera* leave extract; IF/PZQ+MOL, infected mice with *Schistosoma mansonai* treated with praziquantel and *Moringa oleifera* leave extract

**Fig. 5:**
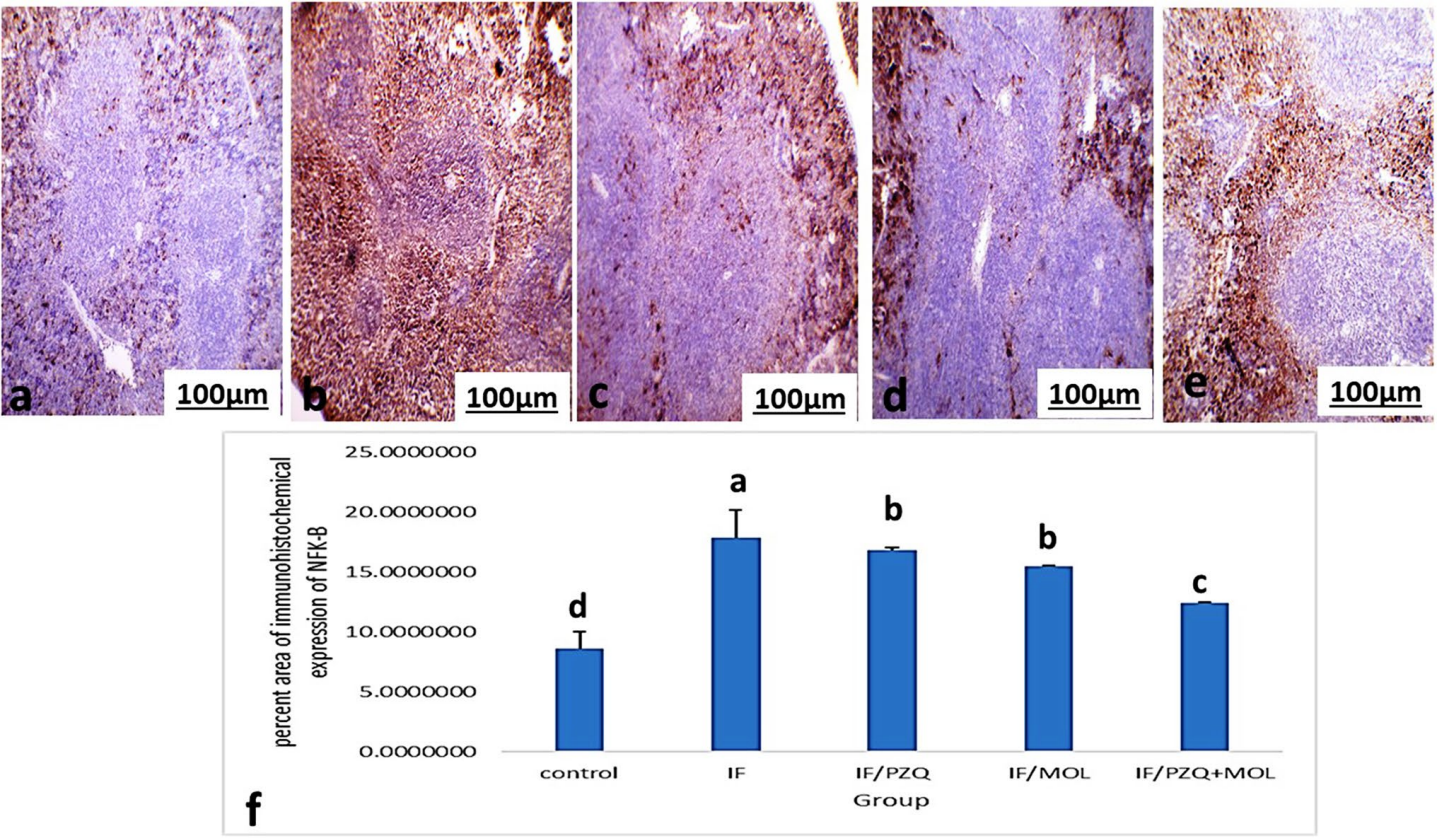
Representative immunohistochemical staining for nuclear factor kappa β (NF-κβ) in the spleen of **a** normal mouse and **b** *S. mansoni*-infected, non-treated mouse. **c** *S. mansoni*-infected, treated mouse with *Moringa oleifera* leave extract (MOL). **d** *S. mansoni*-infected, treated mouse with praziquantel (PZQ). **e** *S. mansoni*-infected, treated mouse with PZQ and MOL (100×). **f** Histogram of the mean percentage areas of NF-κβ, in the spleen of different groups (*n* = 5). Different superscript letters denote significant differences at *P* ≤ 0.05. IF, infected mice with *S. mansoni*; IF/PZQ, infected mice with *Schistosoma mansonai* treated with praziquantel; IF/MOL, infected mice with *Schistosoma mansonai* treated with *Moringa oleifera* leave extract; IF/PZQ+MOL, infected mice with *Schistosoma mansonai* treated with praziquantel and *Moringa oleifera* leave extract

When comparing the percentage-positive cells, within the liver and spleen, between control, IF and IF-treated groups, Figs. 4 and 5 showed a statistically significant difference between groups according to the immunohistochemical expression of NF Kβ in the liver and spleen. IF group possessed the highest mean number of NF-Kβ-positive cells, while IF-treated groups showed the significantly lowest mean number of NF-Kβ-positive cells in the liver and spleen.

## Discussion

The potential advantages of MOL aqueous extract have their origin in antiquity, since it is one of the first known plants used for health maintenance and the treatment of a variety of diseases. In recent years, the anthelminthic effect of MOL aqueous extract has little been verified by researchers. So, this study was carried out to confirm and assess the anti-schistosomal potency of MOL extract. HPLC of an aqueous MOL extract showed that regarding standard used indicated the presence of caffeic acid. However, this compound has also been reported in Laura et al. (2021).

The final body weight of infected untreated mice decreased, whereas the liver and spleen weight increased. One of the negative outcomes of schistosomiasis is growth retardation and an increase in organ weight, which might be caused by a disruption in metabolic activity (Stephenson et al. 2000). In mice, *Schistosoma mansoni* infection resulted in egg excretion in the liver by worms residing in the portal and mesenteric veins, resulting in increased liver and spleen weight (Mukendi et al. 2021). The body weight, liver weight, and spleen weight of infected mice treated with MOL extract alone or with PZQ were all close to normal.

In the current study, the number of Schistosoma worms was lower in the infected treatment groups with both PZQ and MOL extract. This finding is consistent with that of Utzinger et al. (2001) and Almanzor et al. (2014) who found that PZQ and MOL declined gradually the Schistosoma worm count. This could be because the adult worms was died after treatment.

The egg burden in the small intestine and liver of infected mice was greater than in infected mice treated with PZQ, MOL, or both. In this regard, the most mature and immature mice were discovered in infected untreated mice. Considering hundreds of Schistosoma eggs laid by each worm pair every day, the increase in the egg count with the chronicity of the infection was expected (Riad et al. 2007).

The histological changes in the liver and spleen of *S. mansoni*-infected untreated and treated mice were the focus of this investigation. Introducing MOL as an anti-schistosomal supplement was an attempt to find an alternative therapy to PZQ. There were no histopathological differences between the control, MOL, and PZQ+MOL groups in the liver tissues of the uninfected groups. The PZQ group differed from the uninfected group in that there was hydropic degeneration at the central vein and portal region. These modifications agreed with Hussein et al. (2017). Infected untreated mice’s liver sections showed inflammatory infiltration, fiber buildup around Schistosoma eggs and worms, and necrotic foci. These changes were in agreement with Soliman and El-Shenawy (2003). As measured by the decrease in the histopathological change generated in the infected liver group, there was a significant difference between the infected untreated group and those infected and treated with PZQ, MOL, or both. The histological abnormalities in the livers of infected mice improved following therapy. Eggs and worms stuck in tissues caused large diameter granulomas, as seen in the current investigation. These findings were similar to those obtained by Riad et al. (2007). In the current investigation, treating infected mice with PZQ, MOL, or both resulted in a substantial drop in worms, a rise in dead worms, and a decrease in granuloma when compared to the comparable untreated infected group. *S. mansoni* infection caused several histological alterations in the spleen, including significant lymphoid necrosis inside the white pulp, as shown in the current findings. PZQ or MOL, or both, did not completely restore the spleen and lymphoid follicles to their natural histological state. The reduced spleen recovery may be attributed to the short duration of infection therapy, while the spleen damage required 3 to 6 months to encourage its recovery (Benya et al. 1995).

Zhang et al. (2019) have found NFK-B activation during Schistosoma infection. NF-Kβ, a reactive oxygen species-sensitive transcription factor, may have a role in the etiology of liver and spleen abnormalities (Nakajima and Kitamura 2013; Gad El-Hak et al. 2022). Wan et al. (2017) interaction between the NF-Kβ and granuloma formation pathways. In the current investigation, NF-Kβ and increased levels in infected untreated liver and spleen were reduced in treated infected mice with PZQ or MOL, or both. There is a lot of evidence that oxidative stress-induced NF-B activation is linked to Schistosoma-induced fibrosis (Almeer et al. 2018). Reduced and blocked NF-Kβ activity might prevent Schistosoma induction of fibrosis (Gong et al. 2018). As a result, lower NF-Kβ expression in treated-infected rats might be implicated in reduced fibrosis and inflammation of the liver and spleen. Based on the findings of this investigation, we hypothesize that the mechanism of liver and spleen histological changes is caused in part by NF-Kβ activation and repression.

Infection with *S. mansoni* induces hepatocellular damage and raises circulating levels of the liver enzymes ALT and AST. Furthermore, it inhibits protein production (Ramez et al. 2021). After S*. mansoni* infection, circulation levels of AST and ALT were dramatically elevated, although total protein and globulins were reduced. Liver damage occurs as well, as evidenced by histological changes and inflammatory fibrotic granulomas caused by egg deposition as well as worms and their poisons. Serum protein and albumin levels may decrease substantially of *S. mansoni* infections caused to malabsorption following excessive egg ejection and intestinal mucosal injury, or reduced synthesis due to hepatic cell injury (El-Lakkany et al. 2012). The administration of PZQ and MOL, particularly the combination therapy, alleviated serum liver damage in the current investigation. Almanzor et al. (2014) discovered that MOL therapy significantly lowered serum ALT, AST, and ALP activity in *S. mansoni*-infected mice, which is compatible with our findings. Almanzor et al. (2014) revealed that serum ALT, AST, and ALP activities were significantly reduced after treatment of *S. mansoni*-infected mice with MOL, which is consistent with our results. Almanzor et al. (2014) and Abdel Fattah et al. (2020) attributed the protective effects of MOL against liver damage to the phenolic compounds present in its constituent.

## Conclusion

The current data highlight that *S. mansoni* infection caused hepatic and splenic damage in mice. The combination of PZQ and MOL treatment is a new promising natural approach for minimizing pathological alterations in the liver and spleen following *S. mansoni* infection, as evidenced by decreased inflammatory marker NF-Kβ immunohistochemical expression and attenuation of histopathological disorders. Future research should focus on the pharmacological potential and the anti-schistosome effect of PZQ and MOL treatment.

## Data Availability

Data supporting findings are presented within the manuscript.
